# Evaluation of the FecalSwab for Stool Specimen Storage and Molecular Detection of Enteropathogens on the BD Max System

**DOI:** 10.1128/JCM.00178-20

**Published:** 2020-08-24

**Authors:** Melissa Richard-Greenblatt, Candy Rutherford, Kathy Luinstra, Ana María Cárdenas, Xiaoli Lilly Pang, Padman Jayaratne, Marek Smieja

**Affiliations:** a Department of Pathology and Molecular Medicine, McMaster University, Hamilton, Ontario, Canada; b Hamilton Regional Laboratory Medicine Program, St. Joseph’s Healthcare, Hamilton, Ontario, Canada; c Infectious Disease Diagnostics Laboratory, Children’s Hospital of Philadelphia, Philadelphia, Pennsylvania, USA; d Department of Pathology and Laboratory Medicine, Perelman School of Medicine, University of Pennsylvania, Philadelphia, Pennsylvania, USA; e Department of Laboratory Medicine and Pathology, University of Alberta, Edmonton, Alberta, Canada; f Public Health Laboratories, Edmonton, Alberta, Canada; Brigham and Women's Hospital

**Keywords:** BD Max system, FecalSwab, enteropathogens, rectal swabs

## Abstract

The FecalSwab system (Copan Italia, Brescia, Italy) is a convenient alternative to bulk stool for the diagnosis of enteric pathogens. Although the U.S. Food and Drug Administration (FDA) approved for transport and culture of enteric bacterial pathogens, the FecalSwab has not been well assessed for its suitability with molecular platforms. In this study, we evaluated the FecalSwab as a specimen type for the BD Max system using the viral and bacterial enteric panels (BD Diagnostics, Baltimore, MD, USA). A total of 186 unpreserved stool specimens were collected and used to prepare matched bulk stool and FecalSwab samples. Performance was equivalent (*P* > 0.48) to bulk stool for all targets when 50 μl of FecalSwab specimen was loaded onto the BD Max assays. As stool specimens are often collected off-site from the clinical microbiology laboratory and require transport, we assessed the stability of stool specimens stored for up to 14 days at 4°C, 22°C, or 35°C to account for varying transportation conditions. Molecular detection for the majority of viral targets (excluding astrovirus) was unaffected (change in cycle threshold [Δ*C_T_*] ≤ 1) by sample storage temperature over the 2-week period; however, detection of enteric bacteria was variable if specimens were not refrigerated (22°C or 35°C). By demonstrating equivalent performance to matched bulk stool and maintaining molecular detection sensitivity when stored at 4°C, we suggest that the FecalSwab is a suitable specimen type for enteropathogen diagnostics on the BD Max system.

## Abstract (precis)

The FecalSwab system (Copan Italia, Brescia, Italy) is a convenient alternative to bulk stool for the diagnosis of enteric pathogens. Although the U.S. Food and Drug Administration (FDA) approved for transport and culture of enteric bacterial pathogens, the FecalSwab has not been well assessed for its suitability with molecular platforms. In this study, we evaluated the FecalSwab as a specimen type for the BD Max system using the viral and bacterial enteric panels (BD Diagnostics, Baltimore, MD, USA).

## INTRODUCTION

Rapid molecular multiplex testing has revolutionized enteric diagnostics, enabling timely treatment and prompt public health interventions to reduce the spread of gastrointestinal infections. However, diarrheal disease remains a major cause of morbidity and mortality worldwide (1). One of the barriers to managing diarrheal illness results from difficulties in obtaining a stool specimen from outpatients, especially children, which can ultimately lead to delayed diagnosis and contribute to inappropriate treatment (2). As a solution, rectal swabs have been proposed as an alternative to bulk stool collection for the detection of enteric pathogens (3–5).

Although dry flocked rectal swabs have demonstrated equivalent performance to paired unpreserved bulk stool in the molecular detection of enteropathogens (3), it remains necessary to maintain bacterial viability in the preanalytic phase for antimicrobial susceptibility testing and serological typing. The FecalSwab system (Copan Italia, Brescia, Italy) incorporates a flocked swab in a compact liquid-based system (2 ml of modified Cary-Blair medium), providing a convenient alternative to preserved bulk stool for both culture and molecular-based diagnostics. However, the FecalSwab system is only FDA approved for the transport and culture of enteric bacterial pathogens, and it has not been well evaluated for its suitability with molecular diagnostic platforms.

The BD Max system (BD Diagnostics, Baltimore, MD, USA) represents an ideal platform for integrating a FecalSwab specimen type due to its processing design and enteric diagnostic portfolio. The automated platform incorporates both nucleic acid extraction and real-time PCR and provides results for up to 24 samples in less than 3 h. Its enteric solutions portfolio includes a targeted enteric bacterial panel (EBP) and enteric viral panel (EVP) approach. Collectively, these panels provide broad coverage of key bacterial (*Salmonella* spp., *Shigella* spp./enteroinvasive Escherichia coli [EIEC], *Campylobacter* spp., and Shiga toxin-producing organisms such as Shiga toxin-producing E. coli [STEC] and Shigella dysenteriae) and enteric viral pathogens (norovirus, rotavirus, adenovirus, sapovirus, and astrovirus).

In the current study, we evaluated the Copan FecalSwab as a collection/transport device for the molecular detection of viral and bacterial enteropathogens using the BD Max system. As the FecalSwab contains a diluted specimen, we determined equivalent loading amounts to match bulk stool inoculation (standard of care) for the EBP and EVP assays. Once volume optimization was complete, matched FecalSwab and bulk stool clinical specimens were analyzed to evaluate the performance of the FecalSwab as a collection device for the BD Max system. Stability studies were also performed to assess the potential of the FecalSwab to preserve nucleic acid in stool specimens for molecular enteric diagnostics.

## MATERIALS AND METHODS

### Specimen collection.

Experiments were performed using residual unpreserved stool specimens previously characterized using laboratory-developed multiplex PCR methods by the clinical microbiology laboratory. Characterization as positive for one of the targets by the laboratory-developed enteric assay was based on the presence of a signal within 40 cycles. Samples were excluded if the stool was solid (did not take shape of the storage container) or if there was limited stool remaining following standard-of-care testing. Only one specimen per patient was enrolled for testing. Specimen enrollment in the study occurred in two stages as follows: prospective enrollment that included specimens stored at 4°C with a <48-h delay between clinical testing and analysis for the study (*n* = 104) or retrospective enrollment, which we defined as biobanked clinical specimens stored at −80°C (*n* = 82). Prospective enrollment occurred from January to June 2019 through the Hamilton Regional Laboratory Medicine Program (HRLMP; St. Joseph’s Healthcare, Hamilton, Canada).

All specimens were collected through the HRLMP, except those for sapovirus and astrovirus (Children’s Hospital of Philadelphia, USA; Public Health Laboratories, AB, Canada), as these tests were not offered on-site at the time of study. Specimens positive for sapovirus (*n* = 21) and astrovirus (*n* = 21) were previously characterized using laboratory-developed multiplex PCR methods and biobanked at −80°C prior to being shipped to St. Joseph’s Healthcare Hamilton for further processing. All specimens for the study were processed following protocols approved by the Hamilton Integrated Research Ethics Board (HiREB).

### Sample preparation (FecalSwab and standard of care).

Evaluation of the FecalSwab as a collection device for the BD Max EVP and EBP entailed comparison of matched bulk stool-FecalSwab sample pairs. Consequently, each unpreserved stool specimen was prepared using two separate methods as follows: bulk stool as per the BD Max EVP and EBP protocols and the FecalSwab method.

To simulate specimens received in the clinical laboratory, we prepared bulk stool samples differently for the EVP and EBP assays. Stool specimens for the EVP assay in the laboratory are generally submitted as an unpreserved specimen. Therefore, we followed the specimen preparation pathway for unpreserved specimens according to the BD Max package insert, which includes directly inoculating the sample buffer tube with 5 μl of stool. In contrast, the EBP protocol utilizes preserved stool specimens, and as a result, we transferred 5 ml of stool into a Para-Pak Enteric Plus container (Meridian Bioscience, Cincinnati, OH, USA). A total of 10 μl of preserved bulk stool was used to inoculate the EBP sample buffer tube as described by the package insert.

Preparation of the FecalSwab specimen was performed according to the Copan package insert. Briefly, the specimen was collected by inserting the tip of the flocked swab into the unpreserved stool and rotating it to achieve saturation. The swab was immediately placed into the transport tube containing 2 ml of Cary-Blair and vortexed for 60 s using the multitube vortexer (VX-2500 multitube vortexer; VWR, Mississauga, ON, Canada) to allow the fecal sample to elute in the liquid medium.

All specimens were immediately processed on the BD Max system following preparation of the matched pairs. The sample buffer tube was loaded onto the BD Max system along with a PCR cartridge and an EVP or EBP unitized reagent strip. Quantitative (cycle threshold [*C_T_*] values) and qualitative results for each specimen were recorded.

### Determination of FecalSwab specimen loading equivalency for the BD Max system.

BD Max EVP and EBP sample buffer tubes were inoculated with 10 or 50 μl of FecalSwab specimen prepared from a positive clinical specimen and run in triplicate for each molecular target. Criteria for stool selection included prior reverse transcriptase PCR (RT-PCR) *C_T_* values of 15 to 30 and sufficient volume (∼7 ml) to perform volume optimization studies. All specimens were evaluated within 48 h of arrival in the clinical laboratory with the exception of sapovirus and astrovirus, which were previously biobanked. *C_T_* values of each FecalSwab volume were compared to the standard-of-care inoculation volumes (5 μl EVP or 10 μl EBP) recommended by the manufacturer. In this study, we considered the bulk stool volumes recommended by the manufacturer to have previously been optimized to prevent assay overloading (inhibition) and underloading (decreased clinical sensitivity). Therefore, the volume of FecalSwab sample that yielded thresholds equivalent (no statistical difference) to the standard of care was considered optimal and was used for further testing.

### Evaluation of the FecalSwab system for the transport and storage of stool specimens for viral and bacterial molecular diagnostics.

**FIG 1:**
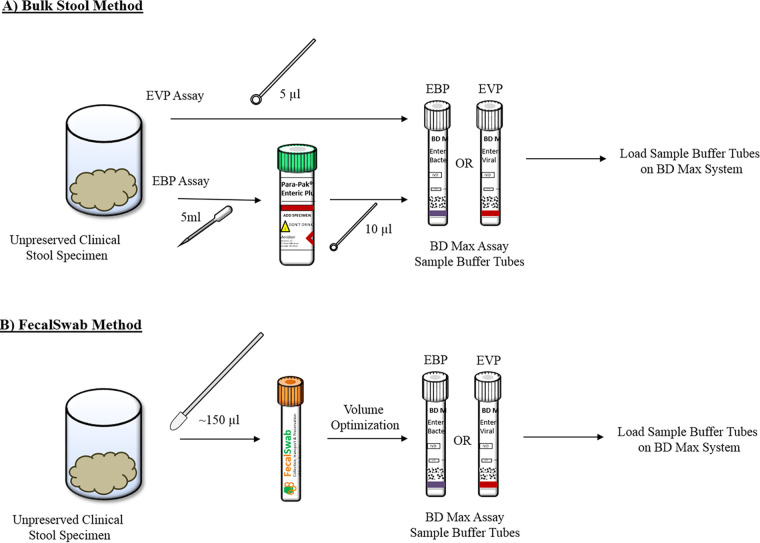
Schematic diagram for sample preparation of bulk stool (A) and FecalSwab (B) methods.

Unpreserved clinical specimens previously characterized as positive for at least one of the pathogen targets were used to prepare FecalSwab samples (according to Fig. 1 protocol) for storage at 4°C, 22°C, and 35°C for 14 days. Clinical specimens were selected based on adequate volume to perform studies (∼2 ml stool) and starting *C_T_* values of <30 to avoid any loss in assay precision when a signal occurred near the limit of detection. The stability of specimens for molecular detection of viral and bacterial enteropathogens was measured using the BD Max EVP and EBP assays. *C_T_* values were collected from FecalSwab triplicates for each condition and target at baseline (immediately following FecalSwab preparation) and compared to 1, 2, 7, and 14 day(s) of storage at 4°C, 22°C, and 35°C.

### Statistical analysis.

Analysis for this study was performed using GraphPad Prism version 7.04 (San Diego, CA, USA). *C_T_* values are summarized as mean ± standard deviation. Sensitivity of the collection method for detecting each enteropathogen target was calculated based on previous RT-PCR characterization by the clinical laboratory. Results that did not conform to those originally identified were considered discordant. McNemar’s test for paired samples was used to assess FecalSwab versus bulk stool detection for each target pathogen. Kappa was calculated to quantify the degree of overall agreement between the two collection methods for each enteric panel.

## RESULTS

### Determination of volume of FecalSwab medium required for equivalent detection to standard of care.

**FIG 2:**
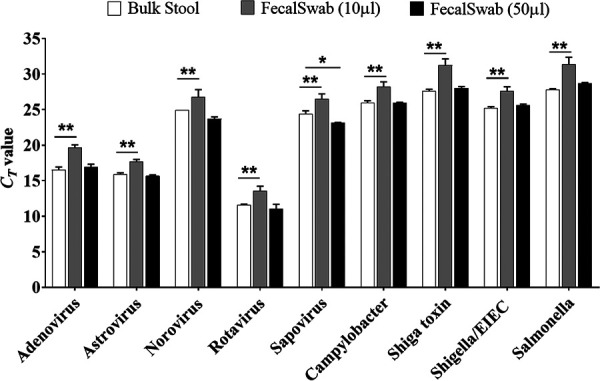
Evaluation of various FecalSwab sample volumes (10 and 50 μl) to achieve equivalent detection to the BD Max standard-of-care (bulk stool) protocols for each EVP and EBP pathogen target. Standard of care recommends 5 μl of unpreserved stool for the EVP assay and 10 μl of specimen preserved in Cary-Blair for the EBP assay. Bars are representative of a single specimen positive for each target presented as mean *C_T_* values of triplicates ± standard deviation (SD). Comparison in molecular detection (*C_T_* values) between bulk stool and 10-μl FecalSwab inoculums for each pathogen target were significant (**, *P* < 0.01). Equivalent detection to bulk stool was found for FecalSwab volumes of 50 μl (*P* > 0.05) for all targets with the exception of sapovirus (*, *P* < 0.05).

The FecalSwab is a more dilute specimen than bulk stool samples; therefore, we first optimized the volume of FecalSwab medium required for equivalent detection in the BD Max EVP and EBP assays. Sample buffer tubes were inoculated with various volumes (10 to 50 μl) of FecalSwab medium, and *C_T_* values were compared to the bulk stool method (Fig. 2). FecalSwab volumes of 50 μl demonstrated equivalent detection to the bulk stool methods for each bacterial and viral target (*P *> 0.05) with the exception of sapovirus, which demonstrated improved sensitivity (*P* = 0.0465). In contrast, lower volumes of FecalSwab inoculum resulted in significantly later *C_T_* values. Therefore, 50 μl of FecalSwab medium was used to perform a clinical evaluation of the FecalSwab protocol and the molecular detection stability studies.

### Clinical evaluation of the FecalSwab for the BD Max EVP and EBP assays.

To validate the FecalSwab for use with the BD Max EVP and EBP assays, we compared the performance of 186 matched FecalSwab-bulk stool specimens in a clinical setting. Of 186 patients, 30 (16.1%) had no pathogen detected, and 156 (83.9%) were positive by at least one of the collection methods for an enteric virus (*n* = 133) or enteric bacteria (*n* = 93). In several cases, multiple pathogens were detected by both collection methods, with 8 samples having two and 1 sample having three pathogens. Therefore, the total pathogen targets (viral and bacterial) detected by the FecalSwab and bulk stool samples yielded 166 and 159 targets, respectively.

The performance of the FecalSwab inoculation compared to bulk stool for each target is summarized in Table 1. Based on the sum of all targets tested for each panel (overall EVP and EBP), overall agreement between the FecalSwab and bulk stool testing protocols was calculated. For the EVP and EBP panels, overall agreement was 99.3% (665/670 targets, κ = 0.97) and 99.5% (372/374 targets, κ = 0.98), respectively, suggesting equivalent detection between the two methods. Of the 7 samples that had discordant results, the FecalSwab specimen was positive for a pathogen target and bulk stool inoculation was negative in all cases.

**TABLE 1:** Clinical evaluation of the FecalSwab as a specimen type for the BD Max enteric viral and enteric bacterial panels

Pathogen target	Results (no.) for indicated collection method				Sensitivity (% [95% confidence interval])^*a*^	
	Total samples positive	Bulk stool positive	Swab positive	Total samples negative	Bulk stool	Swab
Virus (*n* = 133)						
Adenovirus	17	15	17	118	88.2 (63.6–98.5)	100.0 (80.5–100)
Astrovirus	21	21	21	112	100.0 (83.9–100)	100.0 (83.9–100)
Norovirus	20	20	20	113	100.0 (83.2–100)	100.0 (83.2–100)
Rotavirus	24	22	24	111	91.7 (73.0–99.0)	100.0 (85.8–100)
Sapovirus	21	20	21	113	95.2 (76.2–99.9)	100.0 (84.0–100)
Overall EVP	103	98	103	567	95.1 (89.0–98.4)	100.0 (96.5–100)
Bacterium (*n* = 93)						
*Campylobacter*	20	19	20	73	95.0 (75.1–99.9)	100.0 (83.1–100)
*Salmonella*	17	17	17	77	100.0 (80.5–100)	100.0 (80.5–100)
*Shigella*/EIEC	12	12	12	81	100.0 (73.5–100)	100.0 (73.5–100)
Shiga toxin	14	13	14	80	92.9 (66.1–99.8)	100.0 (76.8–100)
Overall EBP	63	61	63	311	95.2 (89.0–99.6)	100.0 (94.3–100)

a Previous RT-PCR characterization for detection of enteropathogens was set as the reference standard for calculation of sensitivity.

We determined the sensitivity of each collection method according to previous stool characterization of target positivity from the clinical laboratory (Table 1). In addition, overall sensitivity for each enteric assay was calculated using the total number of targets detected for the EVP and EBP as the reference for each collection method. The sensitivity of bulk stool for the EVP and EBP assays was ∼95%, whereas the FecalSwab demonstrated 100% sensitivity for both panels. However, McNemar’s test of paired samples did not identify a significant difference in enteric pathogen detection between the FecalSwab and bulk stool collection methods for any of the enteropathogen targets (*P* > 0.05). Therefore, these findings suggest equivalent sensitivity between the two test methods for the detection of enteropathogens on the BD Max system.

Upon further investigation, discordant results were not found to be due to retrospective analysis, as 3/7 discrepant results were prospectively analyzed. In addition, we evaluated the efficiency of internal control amplification in the discordant specimens and observed differences of ≤1 *C_T_* compared to the assay mean *C_T_* for the internal control, suggesting discordant results were not due to RT-PCR inhibition. However, assessment of the FecalSwab *C_T_* values associated with discrepant results revealed the majority of samples to be low positives (mean *C_T_* value = 33.2; range = 28.3 to 36.9) and, therefore, less likely to be reproducible. Nonetheless, these findings suggest that using 50 μl of FecalSwab specimen may provide improved enteropathogen detection in comparison to bulk stool in specimens yielding lower pathogen concentrations.

### Effect of FecalSwab storage time and temperature on the molecular detection of viral and bacterial enteropathogens.

**FIG 3:**
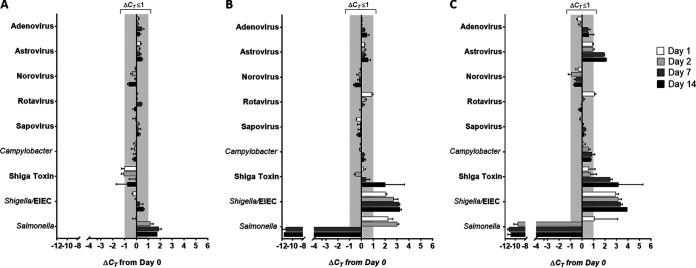
Effect of FecalSwab storage time and temperature on the molecular detection of viral and bacterial enteropathogens in stool specimens. FecalSwab samples were prepared from clinical specimens positive for each pathogen target and stored at 4°C (A), 22°C (B), and 35°C (C) for 14 days. Samples were collected from each FecalSwab at baseline and at days 1, 2, 7, and 14. Cycle threshold (*C_T_*) values were determined using the BD Max EVP and EBP assays. Bars are representative of experimental triplicates and are presented as mean Δ*C_T_* values ± SD from baseline.

As our findings demonstrate the FecalSwab to be a suitable collection device for the BD Max EVP and EBP assays, it was of further interest to evaluate the effect of transport and storage of stool specimens on molecular diagnostic results. We compared the *C_T_* values of clinical stool specimens stored in the FecalSwab at 4°C, 22°C, or 35°C for up to 14 days. Baseline mean *C_T_* values for the majority of enteropathogens ranged from 24 to 27 with the exception of *Salmonella* (*C_T_* = 30.1), rotavirus (*C_T_* = 10.9), astrovirus (*C_T_* = 15.5), and adenovirus (*C_T_* = 9.2). We considered a change in *C_T_* (Δ*C_T_*) score from baseline (day 0) to be equivalent if ≤1 (6) and a Δ*C_T_* of ≥1.1 to be a loss or less than or equal to a −1.1 gain in detection (Fig. 3). Based on these criteria, FecalSwab samples stored at 4°C did not demonstrate any significant change in molecular detection (mean Δ*C_T_* ≤ 1) with the exception of *Salmonella*, which was detected ∼2 Δ*C_T_* later than baseline when measured at days 7 and 14. At higher storage temperatures (22°C and 35°C), we observed a decrease in molecular detection for specific targets over time. Within 24 h of storage at these higher temperatures, there was a significant loss (Δ*C_T_* of ∼3.3) in detection for *Shigella*/EIEC. The change in Shiga toxin detection was not as rapid as *Shigella*/EIEC, as a significant loss in detection was first observed on day 14 at 22°C and on day 7 at 35°C. Astrovirus was the only viral target found to have a significant change in molecular detection, which was only observed at 35°C (Δ*C_T_* ≥ 1 at days 7 and 14).

*Salmonella* exhibited a unique stability profile relative to the other targets measured in this study. In FecalSwab samples stored at room temperature, the number of *C_T_* required to detect *Salmonella* was significantly higher at 24 h (Δ*C_T_*, 2.2 ± 0.7) and at 48 h (Δ*C_T_*, 3.0 ± 0.3) than baseline; however, at days 7 and 14, detection significantly improved (Δ*C_T_* of approximately −11.0). At 35°C, similar results were observed for *Salmonella* with improvements in detection starting on day 2 (Δ*C_T_*, −9.5 ± 1.4). Therefore, viral and bacterial enteropathogens, with the exception of *Salmonella*, can be preserved in the FecalSwab for up to 14 days at 4°C without a significant change in molecular detection. Stool specimens in FecalSwab submitted for viral testing demonstrated stability at higher temperatures (22°C and 35°C) over 2 weeks; however, our findings suggest that the storage and transport at these temperatures for the detection of bacterial enteropathogens should be avoided.

## DISCUSSION

Enteropathogen detection traditionally relies on the collection of bulk stool; however, the inconvenience and time delays associated with attaining this specimen type can result in suboptimum sample return rates and/or delayed and missed diagnostic opportunities. The FecalSwab system represents an alternative method for collecting and transporting stool and rectal swab specimens for enteropathogen detection. The device has the added benefit of transporting fecal specimens in small-instrument-ready tubes, saving space during transport and storage as well as enhancing laboratory workflow by facilitating automated processing.

Copan FecalSwab collection devices have previously demonstrated equivalent performance to bulk stool for enteric pathogen detection using culture methods (7) and with the Cepheid Xpert (6), BioFire FilmArray (8) and BD Max (9) molecular platforms. However, this is the first report evaluating the FecalSwab with the BD Max system EVP assay. In all cases, we observed the sensitivity of the FecalSwab collection method to be 100% when results were compared with previous clinical reporting for the specimen by the laboratory. In contrast, 7 bulk stool specimens did not repeat positive during the evaluation process and were all found to be weak positives based on the *C_T_* values obtained from the matched FecalSwab specimen. As the superior sensitivity of the FecalSwab was not target specific, we attributed these observations to variations in stool viscosity, leading to subtle variations in swab collection volume from each specimen. For example, increased viscosity likely resulted in excess stool picked up by the FecalSwab, thereby increasing sensitivity of the collection method. Therefore, these findings emphasize the importance of identifying a FecalSwab sample testing volume that preserves assay sensitivity for various stool consistencies without overloading or underloading the assay. As we did not observe any loss in sensitivity for the FecalSwab as part of the clinical evaluation, we concluded 50 μl of FecalSwab transport medium (Cary-Blair) to be the optimal sample volume for the BD Max EVP and EBP assays. These findings support those previously published for the BD Max EBP and extended EBP assays (9).

One important consideration when evaluating the implementation of a collection device is the stability of the specimen in context of its use. Few published studies have evaluated the effect of time and temperature on the molecular detection of viral enteropathogens in clinical specimens; however, a study quantifying loss of norovirus RNA RT-PCR positivity in clinical stool samples did not observe a significant change in molecular detection over 7 years of storage at refrigeration temperatures (10). Although environmental factors outside of the host can negatively impact the integrity of viral nucleic acids, the clinical matrix (stool or vomit) by which the virus is shed has shown to be protective (11, 12). When we assessed stability of clinical stool specimens stored in FecalSwab samples for molecular detection, we observed that viral targets remain relatively stable across the tested temperature range up until the final time point (day 14) with the exception of human astrovirus. As a loss in molecular detection was only observed at 35°C on day 7 and 14, these findings suggest a potential role of stool microbiota in the stability of astrovirus nucleic acid. For example, an inverse correlation between the relative abundance of the bacterial genus *Blautia* in stool samples and the effect of that sample on human astrovirus viability has previously been observed (12). It would be of interest to further investigate whether decreased astrovirus stability at elevated temperatures can be replicated in other stool specimens and if stability is associated with certain bacterial populations. Nonetheless, these results suggest that the FecalSwab is a reliable stool specimen transport and storage device for the molecular detection of viral enteropathogens in a wide range of clinical settings.

In contrast to viral enteropathogens, storage temperature had a clear impact on specimen preservation for the molecular detection of bacterial targets. In a previous study, when stored at 4°C, the FecalSwab was found to inhibit the growth of commensal flora while preserving enteric pathogen viability; however, when stored at room temperature, the commensal burden increased by 144.1% in 24 h (7). This overgrowth is likely to have a negative impact on the viability of enteropathogens, especially fastidious organisms, as gastrointestinal flora can directly inhibit intestinal pathogens by competing for nutrients or by inducing the production of inhibitory substances (13). Interestingly, despite being a fastidious microaerophile, Campylobacter jejuni was the bacterial target least affected by changes in FecalSwab storage conditions. These organisms are rarely recovered by culture from FecalSwab samples after 24 h of storage (7, 14), but in the presence of unfavorable environmental conditions, C. jejuni can enter a viable but nonculturable state (15). We suggest that this physiological state permits C. jejuni to persist in stool specimens, which results in its molecular detection being unaffected by FecalSwab storage conditions.

In contrast to other enteric pathogens included in our stability studies, we observed the molecular detection of *Salmonella* to improve over time with increasing temperatures. Earlier work assessing the viability of enteric pathogens from stool stored in Copan FecalSwab samples described significant growth (>1 log CFU/ml) of Salmonella enterica serovar Typhimurium but reduced recovery of other pathogens (Shigella flexneri, Escherichia coli, and Campylobacter jejuni) when specimens were stored at room temperature for 48 h (7). Several studies have provided evidence of multiple mechanisms employed by *Salmonella* that enable the organism to outgrow members of the microbiota under the inflammatory conditions induced by the innate immunity (16). Although we did not evaluate viability in our study, the variability in molecular detection is likely reflective of the growth behaviors of enteropathogens and their interaction with commensal bacteria in stool.

Our study has limitations. Despite having included some of the most common causes of acute diarrhea, we did not cover all pathogens that would be of clinical importance to isolate from FecalSwab specimens. Rojas et al. (9) enhance our findings, as the authors evaluated the compatibility of the FecalSwab with additional pathogens (Yersinia enterocolitica, enterotoxigenic Escherichia coli, *Vibrio* spp., and Plesiomonas shigelloides) using the BD Max extended EBP panels; however, there are still several pathogens, especially intestinal parasites, that require investigation. Another limitation of our study is due to the probability that our findings may not be generalizable to all stool specimens, as we did not evaluate the role of host (microbiota, immune status, and antimicrobial treatment) and pathogen (pathogenicity, metabolism, etc.) factors on molecular detection in our stability studies. Lastly, the current study focuses on the performance of the FecalSwab in its compatibility with the BD Max system. Although our findings and those from others suggest that the FecalSwab can be used for enteric diagnostics with a range of platforms (6, 8, 9), volume standardization and clinical evaluation is required for laboratories using other nucleic acid amplification systems.

One of the major reasons for introducing the FecalSwab system into the clinical setting is for the collection and transportation of rectal swabs. In comparison to direct stool swabbing used in this study, rectal swabs collect smaller volumes of fecal material creating concern that these specimens could have decreased diagnostic sensitivity, especially when further diluted in the FecalSwab transport system. However, rectal swabs are designed to sample beyond the anal canal at the columnar epithelium where the majority of enteropathogens reside. When appropriately collected, direct sampling of the rectum does not only lead to a potentially greater pathogen yield but also enables the collection of mucosal adherent organisms (suggestive of a pathogenic role). In contrast, bulk stool predominately consists of contents derived from the small intestine resulting in a pathogen-diluting effect of the sample. Several studies have demonstrated rectal swabs to have equivalent or greater sensitivity relative to bulk stool in the molecular detection of viral, bacterial, and parasitic enteric pathogens (3, 4, 17–19). Although requiring further validation, results from this study and others (19) support the use of rectal swabs as a specimen type for the detection of enteric pathogens using the BD Max system.

In conclusion, our findings suggest that the Copan FecalSwab is a suitable collection device for both viral and bacterial enteric pathogens on the BD Max system. With increasing consolidation of microbiology laboratories and the requirement to perform testing off-site in centralized laboratories, there was a need to evaluate the utility of the FecalSwab for the transport and storage of stool specimens under various temperatures. However, our findings emphasize the need for a continuous cold chain from time of sample collection to testing for bacterial enteropathogens, which is not always available. Nonetheless, the FecalSwab system represents a valuable tool that pairs the ability to culture enteric bacteria for susceptibility testing (7, 14, 20) and serotyping with molecular diagnostics using the BD Max system. Integrating point-of-care specimen collection methods for diarrheal illness has the potential to improve sample return rates and expedite diagnosis, ultimately leading to improved patient outcomes and the initiation of appropriate infection control measures.
